# The Sanitation of Work-Shops and Public Conveyances

**Published:** 1896-05

**Authors:** B. Merrill Ricketts

**Affiliations:** Cincinnati


### The Sanitation of Work-Shops and Public Conveyances.

BY B. MERRILL RICKETTS, M.D., CINCINNATI.

Read before the Academy of Medicine of Cincinnati, March 16th, 1896.

It would require more time than allotted to do this most important subject justice, consequently but a few passing remarks can be indulged in at this time. The object, however, is to bring a few of the most important features before you for consideration:

### WORK-SHOPS.

1. Kind—whether brick, wood, stone or metal.
2. Location.
3. Ventilation—natural or artificial.
4. Drinking accommodations—filter.
5. Bathing accommodations.
6. Water-closets.
7. Elevators and stairs.
8. Kind of heat—steam, hot air, water, stoves or open fires.
9. Light.
10. Number of square feet to persons.
11. Time to eat.
12. Where eaten.
13. Lounging-places.

All these have great bearing upon those so employed.

### SLAUGHTER-HOUSES AND STOCK-MARKETS.

1. Should not be allowed to exist within the city limits.

2. The sewage from such places should be destroyed by fire or utilized for fertilization, and not allowed to add to the already dangerous contents of sewers.

3. No person under twenty-one years of age should be permitted to work in such places, and especially forbidden to witness the killing of animals.

4. The rendering of lard and tallow should also be prohibited within the corporate limits of a city, town or village.

MARBLE-SHOPS, FOUNDRIES, PLANING-MILLS, FURNITURE FACTORIES,

Or any other having dust should be given special consideration by the sanitarian. Ventilation, baths and lounging-places should be provided, and ample accommodations for changing clothing arranged.

RAG FACTORIES AND WAREHOUSES

Should also be excluded from within the confines of a city, town or village.

Rags, in any quantity, should be subjected to a heat sufficient to destroy any bacteria or parasite. Until such has been accomplished, no bundle of rags, from any locality, should be allowed to be opened or handled as merchandise. Such places should be well ventilated and subjected to the rays of the sun.

Persons under eighteen years of age should not be allowed employment in such places.

SOAP FACTORIES

Are not without their injurious effects, and should constantly be under the inspection of a strict sanitarian, who should forbid their presence in corporation limits.

MALTSTERS AND DISTILLERS

Should locate their factories in country places, and not in a densely populated district. The inhabitants of such places should not be subjected to the vitiated atmosphere as a result of fermentation in such places.

TILE FACTORIES

Should be excluded from the city limits. The law should forbid the employment of persons under eighteen years of age in these factories. They should be under the strictest sanitary regulations and inspected daily. The floors and work-tables should be kept moistened, so that the dust of lead and coloring matter might not infect the atmosphere.

All laborers in and about the place should be compelled to drink during the day a weak solution of sulphuric acid water, which should be kept in earthen jars in various parts of the factory.

Any person manifesting symptoms of lead-poisoning should not be allowed to return to the factory, at any time, to again resume his work.

WHITE LEAD AND PAINT FACTORIES

Should be placed in the same category as tile factories, and governed by the same conditions. The same might be said of brass and copper works and foundries.

MATCH FACTORIES

Have been given more consideration than the others, owing to the certain destruction of the lower jaw of those so employed, but they have not yet been excluded from the corporate limits of cities, towns and villages, which they should be.

BONE CRUSHERS (FERTILIZING ESTABLISHMENTS),

Should also come under the same regulations.

SHOE FACTORIES.

As a rule, these factories have better sanitary accommodations than any other. The nine thousand persons thus employed in this city have very good light, heat and ventilation, and sewer connections.

FURNITURE FACTORIES

Have poor accommodations for their employes, the ventilation and atmosphere being imperfect.

The wood and sand dust are about the worst elements to be found in the atmosphere (so far as solid matter is concerned).

Feathers, hair, shucks, wool and moss should be subjected to a heat sufficient to destroy anthrax, with other contagious, infectious and parasitic life.

CHILDREN

Should be kept in school and not allowed to become engaged at work in any kind of factory before eighteen years of age. It is cheaper, from a financial point of view, to keep them in school until that age than to care for them in alms-houses in declining years, as the result of not having had educational advantages in early life. Besides, one can never be compensated for the loss of a finger, hand, toe, foot or eye, or any of the disfigurements incident to the life of a mechanic. This is especially so with girls. Many boys and girls are forced to adopt vocations entirely different to what they would have done, as a result of the deformity sustained.

The social relations of those so afflicted are more or less impaired. This is more marked with girls. The menstrual period is utterly disregarded, those of their own sex, in places of authority, apparently being the most disregardful. Every woman should seek and find mental and physical quietude and rest at the time of menstruation.

COOKS, DAIRYMEN, WAITERS AND BAKERS

Should be given special consideration; also nurses and those who are continually coming in close contact with people. Gonorrhœa, syphilis and tuberculosis are too common and too easily communicated to be utterly disregarded by the sanitarian. A weekly or monthly inspection by a competent physician might eliminate many of the dangers to which the innocent are subjected.

MONEY.

Money, both paper and coin, should, in the hands of certain persons, be subjected to a sufficient heat to destroy all bacteria and parasites. Banks and institutions handling large amounts should have a receptacle for this purpose. It would cost $25.00, but would save many times that amount in preventing disease, loss of time and death, besides taxation for services rendered to such afflicted.

VEHICLES

Of all kinds, both public and private, should be supplied with automatic brakes. Especially is this desirable in a rough, uneven territory like that surrounding Cincinnati. An automatic "release" should also be attached to all kinds of animal vehicles, so that the tongue and shafts could be disengaged from the vehicle in case of accident. In this way many serious and fatal accidents could be avoided.

All kinds of public conveyances, such as carriages, wagons, omnibuses and chaises, should be under the care of a competent superintendent, whose duty it should be to have them receive a daily cleansing and inspection.

THE BICYCLE,

While common, is one of the most important of vehicles for consideration, because of its injurious effects. These are especially noticeable in children, who, like the adult, should not indulge to excess. The best and quickest way to prevent these excesses is to enlighten the riders as to the dangers. An increased pulse-rate fron 80 or 100 to 160 or 200 is a serious matter.

STREET-CARS.

No straw or matting, except wire or wood, should be on the floor. Floors should be washed twice each day.

All glass in a car should have heavy wire screens on the inside to prevent lacerations from glass in case of collision.

Seats should be wood and not cushions. The backs of seats should also be wood. If cushions are in a car, they should be subjected to dry heat sufficient to destroy all bacteria or parasites.

Lights should be constant, and not allowed to shine forty different ways within as many seconds.

Heat in a car needs as much attention as an engine, and should be placed at one point, where persons are not compelled to sit over it.

Ventilation should be as near perfect as possible. It should be under the control of an intelligent conductor, and not an unintelligent passenger.

No person should stand in a street car.

None but the side entrance at the back end of a street-car should be allowed. A gate should be at this place, and it should not be opened, for either exit or entrance, until the car has come to a standstill. It should be a crime for any one but the conductor to open the gate.

Open summer cars should be provided with heavy iron screens on either side, to prevent arms, legs and heads from protruding beyond the car.

INCLINES TO HILL TOPS.

While but two or three serious accidents have occurred to these during the history of the city, many precautions could be taken that have not been taken to prevent further trouble.

Two motormen should be kept constantly in the lookout, so that in the event of the disability of one from apoplexy, fainting, accident or death, the other could prevent the destruction of life and property.

There should be four wheels on each side of the car, that three would safely carry it in case one should break.

The law provides that the locomotive engineer and fireman shall remain in the cab during the running period of the engine, but does not so provide for the presence of two pilots on watch during the running of the ordinary river steamboat.

I was called to see a prominent pilot who was stricken by cerebral hemorrhage within thir'y minutes after the landing of one of the largest steamers on the Ohio river. She carried two hundred and fifty souls and an immense cargo down the river, passing numerous piers, on one of the stormiest nights of the year. No person was present with him in the pilot house. Here was a man in charge of two hundred and fifty souls and two hundred thousand dollars worth of property, who had upon a previous occasion been likewise afflicted.

Ocean steamers have two men on duty capable of using the compass—the compassman and captain—the first to manipulate the compass under the direction of the latter, whether he be near or far away. But on narrow bodies of water, like the ordinary rivers, accidents in the way of collisions occur quickly.

